# Studies on antidiarrhoeal, antispasmodic and bronchodilator activities of *Operculina turpethum* Linn

**DOI:** 10.1186/1472-6882-14-479

**Published:** 2014-12-12

**Authors:** Huma Shareef, Ghazala H Rizwani, Safur R Mandukhail, Naoharu Watanabe, Anwar H Gilani

**Affiliations:** Department of Pharmacognosy, Faculty of Pharmacy, University of Karachi, University road, Karachi, 75270 Pakistan; Natural Product Research Unit, Department of Biological and Biomedical Sciences, Aga Khan University Medical College, Karachi, 74800 Pakistan; Department of Pharmacy, Quaid-i-Azam University, Islamabad, 45320 Pakistan; Department of Applied Biology and Chemistry, Faculty of Pharmacy, Shizuoka University, 836 Ohya, Shizuoka, 422 Japan; Department of Pharmacy, College of Health Sciences, Mekelle University, Mekelle, Ethiopia

**Keywords:** *Operculina turpethum*, Antidiarrhoeal, Antispasmodic, Bronchodilator, Ca^++^ antagonist

## Abstract

**Background:**

Traditionally, *Operculina turpethum* has been used in a wide range of ailments such as, gastrointestinal disturbances and asthma. It is found in China, South Asia, Pacific Islands, and Australia. This study was aimed to provide a possible pharmacological basis for the medicinal use of *O. turpethum* in gut and airways disorders.

**Methods:**

Castor oil-induced diarrhoeal mice model and isolated tissue preparations such as, rabbit jejunum and guinea-pig tracheal preparations were used to test the antidiarrhoeal, antispasmodic and bronchodilator effects and the possible mode of action(s) of the 70% aqueous-ethanolic extract of *O. turpethum* black variety (OTB).

**Results:**

In the castor oil-induced diarrhoea in mice, the crude extract of OTB caused a dose-dependent (300–1000 mg/kg) protection from diarrhoea, similar to that of loperamide. In isolated rabbit jejunum preparations, OTB produced a dose-dependent inhibition of spontaneous and high K^+^(80 mM)-induced contractions with resultant median effective concentrations (EC_50_ with 95% confidence interval) of 1.04 mg/ml (0.59-1.54) and 0.12 mg/ml (0.10-0.15; n = 4) respectively, thus showing more potency against K^+^. Pretreatment of the tissue with OTB (0.01 and 0.03 mg/ml) caused a rightward shift in the concentration response curves of Ca^++^, similar to that of verapamil. In isolated guinea-pig tracheal preparations, OTB caused inhibition of carbachol and high K^+^-induced constriction at similar concentrations with respective EC_50_ value of 0.66 mg/ml (0.53-0.82) and 0.59 mg/ml (0.45-0.62). Activity-directed fractionation revealed that the ethyl acetate fraction was more potent than the parent crude extract and hexane fraction.

**Conclusion:**

These results suggest that the crude extract of *O. turpethum* possesses antidiarrhoeal, antispasmodic and bronchodilator activities, mediated possibly through the presence of Ca^++^ antagonist like constituent(s), though additional mechanism(s) cannot be ruled out. Thus, this study provides the evidence for the medicinal use of plant in diarrhoea and asthma.

## Background

Diarrhea is defined as increased frequency of loose stools often accompanied with abdominal cramps, which affects over 3 billion people causing approximately 5 million deaths annually. Pakistan stands 5^th^ amongst the countries reporting under-5 deaths due to diarrheal diseases [1]. Diarrhea can either be a manifestation of any chronic disease [2] or due to infectious etiology. In chronic diarrheal diseases, treatment is needed as long as the symptoms last which could be for weeks, months or even lifetime. Management of chronic diarrhea includes anti-motility and/or bulk-forming agents which are associated with distention, bloating, nausea and constipation, particularly in long term use [3]. On the other hand, alternate medicine constituting mainly herbs offers relatively safe and cost-effective treatment [4].

Most of the disorders of respiratory system including asthma result from the hyperactivity of airways. The prevalence of airways disorders has been increased globally, particularly in children. According to the World Health Organization, it affects about 5–10% globally [5]. The available conventional medicine for the hyperactive airways disorders particularly for asthma is neither cost-effective and nor always be safe, therefore, people are looking for the alternative therapeutic measures.

Phytomedicine is one of the most popular alternative remedies, and there is a revival of interest globally in the use of botanicals, and the physicians of the pharmaceutical medicine are now beginning to accept the herbal remedies once they are scientifically validated [4].

*Operculina turpethum* (L.) Silva Manso is a synonym of *Ipomoea turpethum* (L.) R. Br. (*Convolvulaceae)* and commonly known as Indian Jalap or Turpeth. It occurs in two forms namely *sveta* and *krishna* which are commonly known as white and black respectively. It is found in Pakistan, India, Southern China, South East Asia, Pacific Islands, and Australia. Traditionally, its roots and stems have been used in wide range of ailments including gastrointestinal disturbances such as gastric ulcer, diarrhoea, constipation, cough, asthma, splenomegaly, anemia, tumors, raised lipid levels and obesity [6]. Furthermore, it has been used to relieve fevers and to treat paralysis and pain in the joints and muscles [7–10].

Phytochemical studies revealed the presence of different types of bioactive compounds such as, coumarins, turpethin, α and β rahmnose, fructose, scopletin, β- sitosterol, betulin and lupeol. Some triterpenoids were also isolated from it like cysloartenol, lanosta -5- ene and 24-methylene- δ-5- lanosterol. A resinous material which contain turpethinic acids A, B, C, D & E and dammarane-type saponins, operculinosides, glycosides and turpethosides were isolated from the roots of *O. turpethum* [10–12].

The plant has been studied for a few biological activities, such as antioxidant, anticancer [7], hepatoprotective [8] and antidysmenorrhea [13]. It is recommend by traditional healers in various gut ailments instead of other drugs *Exogonium purge* / *I. purga* (True jalap) and *Rheum emodi* (rhubarb) which are used in gastric ailments. However, the plant (black variety) has not been studied pharmacologically for its use in hyperactive gut and airways disorders. Therefore, the current study was aimed to investigate the antidiarrhoeal, antispasmodic and bronchodilator activities of the *O. turpethum* to provide pharmacological basis for its medicinal uses in diarrhoea and asthma.

## Methods

### Plant collection and identification

The root of *O. turpethum* black variety (OTB) was purchased from the local market. After the identification of plant material by Prof. Dr. Ghazala H. Rizwani, Faculty of Pharmacy, University of Karachi, a voucher specimen, # 037a has been deposited in the herbal museum of the Faculty of Pharmacy, University of Karachi.

### Extraction and fractionation

One kilogram of air dried roots of *O. turpethum* black variety (OTB) was coarsely powdered and percolated in 70% ethanol at room temperature for 15–20 days in a separate glass container. Percolate was filtered through Whatman filter paper No. 1 and this process was repeated for three times and then combined all three filtrates. Organic solvent evaporated under reduced pressure and controlled temperature (40°C). Blackish brown thick semi-solid extract of OTB (46.0 g) was obtained that was first partitioned with ethyl acetate (EtOAc) and distilled water (H_2_O) by 1:1 ratio; ethyl acetate and water layers were obtained and a portion of crude extract of OTB was also treated with *n*-hexane (Hex), on vigorous shaking it was yielded to reddish brown precipitates. All these filtrates were evaporated further on rotatory evaporator separately to obtained ethyl acetate (11.65 g) and hexane fractions (5.0 g) of OTB respectively.

### Phytochemical screening

The crude ethanol extract of OTB was tested for the presence of various phytochemical classes of compounds such as anthraquinones, tannins, saponins, flavonoids, terpenoids, alkaloids, ketones, sugars, glycosides, steroids and amino acids using previously described method [14].

### Drugs and standards

The following reference chemicals were obtained from the sources specified: acetylcholine chloride, atropine sulphate, carbachol, verapamil hydrochloride potassium chloride (Sigma Chemical Company, St. Louis, MO, U.S.A.) and castor oil (Karachi chemical Industries, Karachi, Pakistan).

The following chemicals were used to make the physiological salt solutions: glucose, magnesium chloride, magnesium sulphate, potassium dihydrogen phosphate, sodium bicarbonate, sodium dihydrogen phosphate, (E. Merck, Darmstadt, Germany), calcium chloride (Sigma Chemical Company) and sodium chloride (BDH Laboratory Supplies, Poole, England).

All chemicals used were of the highest purity grade available. Stock solutions of all the chemicals were made in distilled water and the dilutions were made fresh on the day of the experiment.

### Animals

The local breed of rabbits (1.5-2 kg), guinea pig (450–550 g) and Balb-c mice (20–25 g) of either sex were used in the study. The animals were housed in the animal house of the Aga Khan University Karachi under controlled temperature (23-25°C), kept in plastic cages (47 × 34 × 18 cm^3^) with sawdust (renewed after every 48 h). Animals were given tap water *ad libitum* and a standard diet and were fasted for 24 h before the experiment and sacrificed by cervical dislocation. Experiments performed with the rulings of the Institute of Laboratory Animal Resources, Commission on Life Sciences, National Research Council [15] and this study was part of the PhD proposal of Dr. Huma Shareef, approved by the Board of Advanced Studies and Research, University of Karachi, Karachi, Pakistan.

### *In vitro*experiments

#### Rabbit jejunum

The spasmolytic activity and possible mode of action of the plant materials were studied by using isolated rabbit jejunum as described previously [16, 17]. The animals had free access to water but were fasted for 24 h before the experiment. After cervical dislocation, the abdomen was cut open and the jejunum portion isolated out. Each segments of rabbit jejunum of about 2–3 cm length, was mounted in a 10 ml tissue bath, containing Tyrode’s solution, maintained at 37°C and aerated with carbogen (95% O_2_ and 5% CO_2_). Tension changes in the intestinal tissue were recorded via a force displacement transducer (model FT-03) coupled with bridge amplifier and PowerLab 4/25 data acquisition system connected to computer running LabChart 6 software (ADInstrument, Sydney Australia). The tissues were allowed to equilibrate for at least 30 minutes at preload of 1 g before the addition of any drug, during which the tissue was washed with fresh fluid at an interval of every 10 minutes.

Rabbit jejunum exhibits spontaneous contractions under these experimental conditions, thus allowing to test the spasmolytic effect of the test materials without using an agonist [18]. To assess whether the spasmolytic activity of the test substance was mediated through Ca^++^ channel blockade (CCB), the high K^+^ (80 mM), as KCl, was used to depolarize the preparations [19]. The high K^+^ (80 mM) was added to the tissue bath, which produced a sustained contraction. The test materials were then added in a cumulative fashion to obtain concentration-response curves (CRCs) for inhibitory effect. To confirm the Ca^++^ antagonist property of the test substance, the tissue was allowed to stabilize in normal Tyrode’s solution, which was then replaced with Ca^++^ -free Tyrode’s solution containing EDTA (0.1 mM) for 30 minutes in order to remove Ca^++^ from the tissues. This solution was further replaced with K^+^-rich and Ca^++^-free Tyrode’s solution. Following an incubation period of 30 minutes, Ca^++^ CRCs were obtained. When the control Ca++ CRCs were found super-imposable (usually after two cycles), the tissue was pre-treated with the crude extract for 60 minutes. The CRCs of Ca^++^ were then reconstructed in the presence of different concentrations of the extract.

#### Guinea-pig trachea

Trachea was dissected out from guinea pigs and kept in normal Kreb’s solution. The tracheal tube was cut into rings, 2–3 mm wide, each containing about two cartilages and then tracheal ring strips were mounted in a 20-ml tissue bath containing Kreb’s solution, maintained at 37°C and aerated with carbogen gas [20]. The composition of Kreb’s solution was (mM): NaCl 118.2, NaHCO_3_ 25.0, CaCl_2_ 2.5, KCl 4.7, KH_2_PO_4_ 1.3, MgSO_4_ 1.2 and glucose 11.7 (pH 7.4). A tension of 1 g was applied to each tracheal strip and was kept constant throughout the experiment. The tissues were allowed to equilibrate for at least 45 minutes before the addition of any drug. Carbachol (CCh;1 μM) and K^+^ (80 mM) were used to induce sustained contractions and the relaxant activity of crude extract was assessed by adding in a cumulative fashion. The changes in the tissue responses were recorded on a force-displacement transducer (model FT-03) coupled to a bridge amplifier and PowerLab 4/25 data acquisition system connected to computer running LabChart 6 software (ADInstrument, Sydney Australia).

### *In vivo*experiments

#### Castor oil-induced diarrhoea

Antidiarrhoeal activity was studied in mice as described previously [21, 22]. Animals were fasted for 24 h before the experiment, housed in individual cages and divided into six groups, each containing five mice. The first group received saline (10 ml/kg, p.o.) and served as a negative control. The doses of the test extract were selected on trial basis and three increasing doses were given orally. A group of mice was treated with loperamide (10 mg/kg, p.o.), as a positive control. One hour after treatment, each animal received 10 ml/kg of castor oil orally through a feeding needle. Up to 4 h after the castor oil challenge, the presence of diarrhoeal droppings was noted on blotting sheets in the individual cages. The percent protection against the castor oil-induced diarrhoea was calculated based on the number of faeces in each cage.

#### Acute toxicity

Balb-C mice (20–25 g) were divided into different groups containing 5 mice in each. The test was performed using increasing doses (1, 3 and 5 g/kg) of the plant extracts, given orally, in 10 ml/kg volume to different groups serving as test groups. Another group of mice was administered saline (10 ml/kg) serving as the negative control. The mice were allowed food *ad libitum* during a 24 h and kept under regular observation for mortality and toxic effects as gastrointestinal spasms, anorexia, diarrhoea and behavioural changes.

#### Data analysis and statistics

The data are expressed as mean ± SEM and the median effective concentrations (EC_50_) values are given as geometric mean with 95% confidence intervals (CI). The CRCs were analyzed by non-linear regression (Sigmoidal dose–response curve variable slope). One-way Analysis of Variance (one-way ANOVA) followed by Tukey’s post-test was used to determine the significant difference among the pairs. P-value less than 0.05 (P < 0.05) was considered statistically significant. All the graphs, calculation and statistical analyses were performed using GraphPad Prism software version 4.00 for Windows, (GraphPad Software, San Diego California USA, http://www.graphpad.com).

## Results

### Phytochemical screening

Qualitative phytochemical study of the crude extract of OTB showed the presence of tannins, saponins, terpenoids, alkaloids, sugars, glycosides and steroids.

### Spasmolytic effect of *O. turpethum*black variety

**Figure 1:**
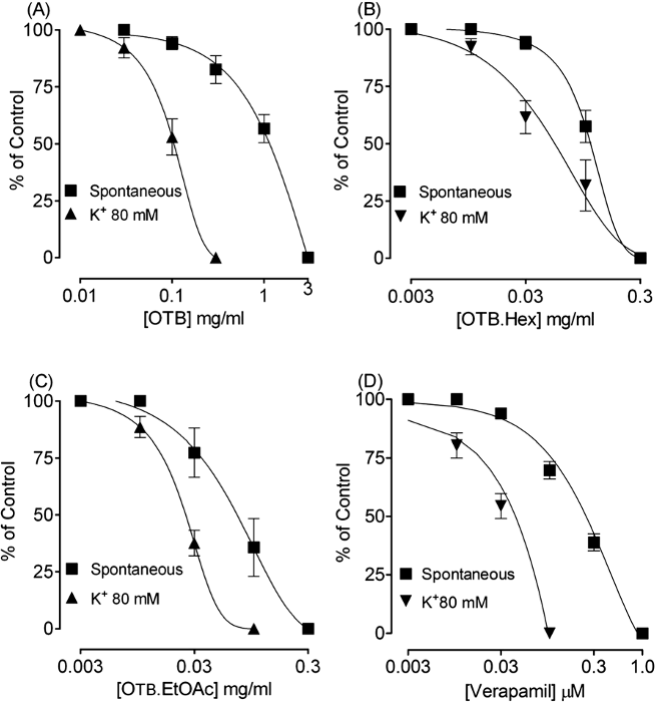
**Concentration-response curves showing the effect of the crude extract of** ***O. turpethum*** **black variety (OTB) (A), and its hexane fraction (OTB.Hex) (B) and ethyl acetate fraction (OTB.EtOAc) (C) and compared with that of verapamil (D) on spontaneous and high K** ^**+**^ **-induced contractions in isolated rabbit jejunum preparations.** The symbols represent mean ± S.E.M of 4 to 6 determinations.

In isolated rabbit jejunum preparations, the crude extract of OTB, inhibited the spontaneous contractions in a concentration-dependent manner with resultant EC_50_ value (95 CI) of 1.04 mg/ml (0.59-1.54; n = 4). When tested against the high K^+^ (80 mM)-induced contractions, OTB caused a relaxant effect at lower concentrations with resultant EC_50_ value of 0.12 mg/ml (0.10-0.15; n = 4) as shown in Figure 1A. Similar pattern of inhibitory effect was seen with verapamil, which produced relaxation of K^+^ (80 mM)-induced contractions at lower concentrations with EC_50_ value of 0.03 μM/ml (0.02-0.04; n = 4) as compared to that obtained on spontaneous contractions with EC_50_ value of 0.24 μM/ml (0.22-0.25; n = 4), as shown in Figure 1D.`

The hexane fraction of OTB (OTB.Hex) also caused a dose-dependent inhibition of spontaneous and high K^+^-induced contraction, with respective EC_50_ values of 0.12 mg/ml (0.10-0.15; n = 4) and 0.063 mg/ml (0.03-0.13; n = 4), thus, showing a greater potency against K^+^, similar to what was observed with verapamil as shown in Figure 1B.

The ethyl acetate fraction of OTB (OTB.EtOAc) was more potent than the crude extract and hexane fraction in its inhibitory effect, with resultant EC_50_ values of 0.07 mg/ml (0.03-0.12; n = 4) and 0.03 mg/ml (0.01-0.03; n = 4) against spontaneous and high K^+^-induced contractions respectively (Figure 1).

**Figure 2:**
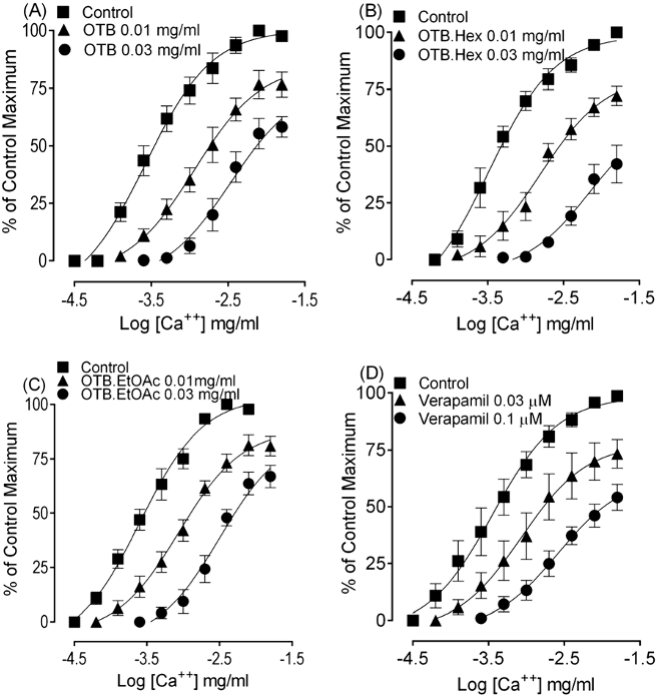
**The concentration-response curves of Ca** ^**++**^ **in the absence and presence of the increasing concentrations of the crude extract of** ***O. turpethum*** **black variety (OTB) (A), hexane fraction (OTB.Hex) (B), ethyl acetate fraction (OTB.EtOAc) (C) and verapamil (D) constructed in Ca** ^**++**^ **-free and K** ^**+**^ **-rich (80 mM) Tyrode’s solution in isolated rabbit jejunum.** The symbols represent mean ± S.E.M, n = 4.

To confirm the Ca^++^ channel blocking activity of the OTB, the Ca^++^concentration curves were constructed in the K^+^ rich and Ca^++^-free medium in the absence and presence of test material. The parent extract and its organic fractions showed concentration-dependent rightward shifts in the Ca^++^ CRCs, similar to that of verapamil, a standard Ca^++^ channel blocker as shown in the Figure 2.

### Bronchodilator effect of *O. turpethum*black variety

**Figure 3:**
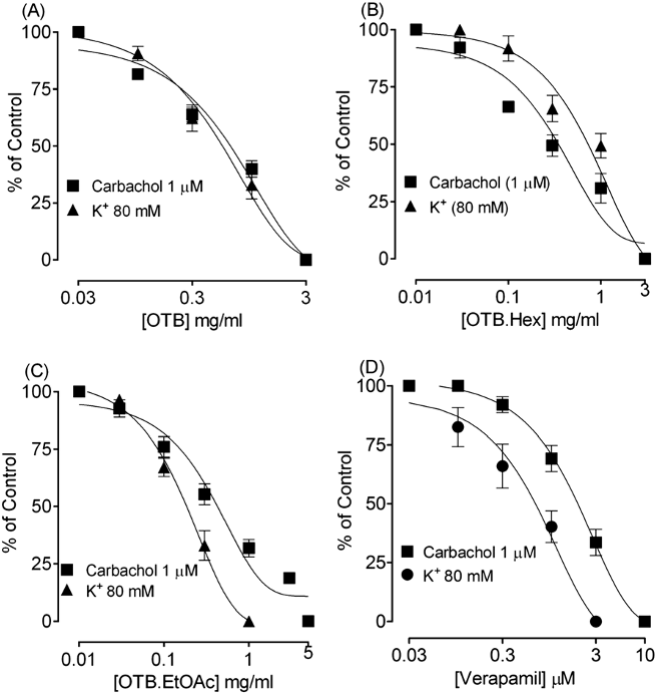
**Concentration-response curves showing the inhibitory effect of the crude extract of** ***O. turpethum*** **black variety (OTB) (A), hexane fraction (OTB.Hex) (B), ethyl acetate fraction (OTB.EtOAc) (C) and verapamil (D) on carbachol (1 μM)- and high K** ^**+**^ **-induced contractions in isolated on guinea-pig tracheal preparations.** The symbols represent mean ± S.E.M, n = 4.

In isolated guinea-pig tracheal preparations, OTB produced a concentration-dependent inhibition of CCh and K^+^-induced contractions with respective EC_50_ value of 0.66 mg/ml (0.53-0.82; n = 4) and 0.59 mg/ml (0.45-0.62; n = 4) as shown in Figure 3A. Verapamil also caused a dose-dependent inhibition of both spontaneous and high K^+^-induced contractions (Figure 3D).

The hexane fraction of OTB (OTB.Hex) also caused inhibition of the carbachol and high K^+^-induced contractions with resulting EC_50_ values of 0.61 mg/ml (0.29 - 1.10; n = 4) and 0.71 mg/ml (0.29 - 1.10; n = 4) respectively (Figure 3B).

Like the parent crude extract and hexane fraction, the ethyl acetate fraction of OTB (OTB.EtOAc) caused a dose-dependent inhibition of high K^+^ and carbachol-induced contractions with resultant EC_50_ values of 0.19 mg/ml (0.24-0.13; n = 4) and 0.44 mg/ml (0.69-0.25; n = 4) respectively, a pattern similar to that of verapamil as shown in Figure 3C.

### Antidiarrhoeal effect of *O. turpethum*black variety

**Figure 4:**
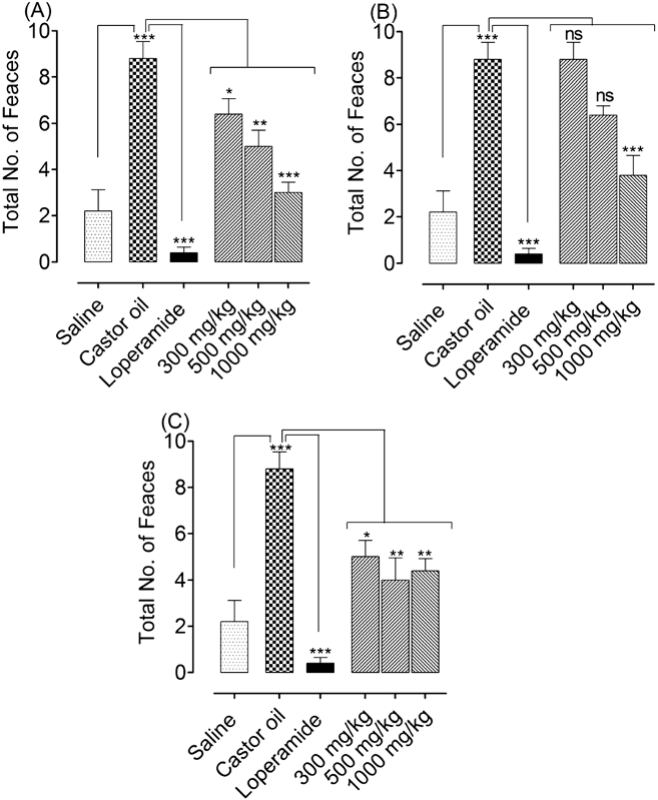
**Bar diagrams showing the dose-dependent antidiarrhoeal effect of the crude extract of** ***O. turpethum*** **black variety (OTB) (A), and its hexane fraction (OTB.Hex) (B) and ethyl acetate fraction (OTB.EtOAc) (C) in castor oil-induced diarrhoea in mice.** The symbols represent mean ± S.E.M, n = 5. One-way ANOVA followed by Tukey’s post-test. *P < 0.05; **P < 0.01 and ***P < 0.001 show a comparison of saline group to caster oil group and caster oil group to drug treated groups.

Based on the medicinal use of *O. turpethum* in hyperactive gut disorders, such as diarrhoea and spasm, its aqueous methanol crude extract and its hexane and ethyl acetate fractions were tested for the possible antidiarrhoeal effect in mice. The crude extract, hexane and ethyl acetate fractions of OTB, like loperamide significantly inhibited the frequency of defecation compared with the untreated group (i.e. mice receiving neither OTB and its fractions, nor loperamide, but castor oil only). All test materials at the maximal tested dose (1000 mg/kg) reduced the faecal droppings and provided 66%, 57% and 50% protection respectively, while 96% protection was shown by loperamide (10 mg/kg), as shown in Figure 4.

### Acute toxicity

The acute toxicity testing of OTB in mice was conducted at different doses (1, 3 and 5 g/kg); there was no mortality or changes in gross behavior up to the dose of as high as 5 g/kg, indicating that the OTB is relatively safe up to the maximum tested dose, which is distinctly higher than those used therapeutically.

## Discussion

The OTB was tested for its possible antidiarrhoeal effect in mice and the result showed that OTB controlled castor oil-induced diarrhoea significantly like loperamide, a standard antidiarrhoeal agent [23]. Castor oil induces diarrhoea in mice due to the action of ricinoleic acid formed by the hydrolysis of the oil [24], and produces changes in the transport of water and electrolytes resulting in a hyper-secretory response and generation of giant contraction of the intestine [25]. Thus, a potential antidiarrhoeal agent may exhibit its antidiarrhoeal effect by inhibiting gut motility and/or electrolyte out flux (diarrhoeal droppings). The protective effect of the OTB against the castor oil-induced diarrhoea in mice, similar to loperamide, suggests that it has either an inhibitory effect on gut motility or on electrolyte out flux. To see its possible inhibitory effect on gut motility, the OTB was further studied in the *in vitro* experiments.

The spontaneously beating isolated rabbit jejunum preparations were used to test a possible inhibitory (spasmolytic) effect of test substances without the use of a spasmogen [26], where OTB caused a concentration-dependent inhibition of spontaneous contractions, thus showing antispasmodic action. The contraction of smooth muscle preparations, including rabbit jejunum, is dependent upon an increase in the cytoplasmic free calcium (Ca^++^), which activates the contractile elements [27]. The increase in intracellular Ca^++^ occurs either via influx through voltage-dependent Ca^++^ channels (VDCs) or its release from intracellular stores in the sarcoplasmic reticulum. Periodic depolarization and repolarization regulates the spontaneous movements of the intestine and at the height of depolarization, the action potential appears as a rapid influx of Ca^++^ via VDCs [28]. Thus, the inhibitory effect of the OTB on spontaneous movements of rabbit jejunum may appear to be due to a CCB effect mediated possibly due to interference of Ca^++^ influx through VDCs. The presence of Ca^++^ antagonist constituent(s) was further confirmed when pre-treatment of the tissue with plant extracts shifted the Ca^++^ CRCs to the right, similar to that caused by verapamil.

In view of the medicinal use of the plant in airways disorders, such as asthma, OTB was further studied in isolated guinea-pig tracheal preparations for the possible bronchodilator effect. Like in the gut preparation, OTB produced a concentration-dependent inhibition of carbachol (CCh) and high K^+^-induced contractions, similar to that of verapamil indicating bronchodilator effect mediated possibly through Ca^++^-antagonist effect [29]. Activity-directed fractionation revealed that both ethyl acetate and hexane fractions, inhibited carbachol- and high K^+^-induced contractions like in gut preparation, indicating that the active constituents mediating antispasmodic and bronchodilatory effect are widely distributed.

The presence of flavonoids, saponins and tannins, revealed by preliminary phytochemical analysis, support the CCB effect of the plant extract because plant derived flavonoids, tannins [30] and saponins [31] have been found to possess CCB effect, which might be responsible for its medicinal use in diarrhoea, gut spasm and asthma, though additional mechanism(s) cannot be ruled out.

In acute toxicity studies, OTB did not cause any mortality up to the tested dose of as high as 5 g/kg, which is much higher than the routinely used dose. However, more detailed sub-acute and chronic toxicity studies are required to establish the safety of this commonly used plant.

## Conclusion

This study shows that the antidiarrhoeal, antispasmodic and bronchodilatory activities of the crude extract of *Operculina turpethum* (black verity) and its organic fractions are mediated through the blockade of Ca^++^ channels; thus, this study provides a possible pharmacological base to its medicinal use in diarrhoea, gut spasms and asthma, though additional mechanism(s) cannot be ruled out.
